# Biosynthesis of Bacterial Cellulose/Carboxylic Multi-Walled Carbon Nanotubes for Enzymatic Biofuel Cell Application

**DOI:** 10.3390/ma9030183

**Published:** 2016-03-09

**Authors:** Pengfei Lv, Quan Feng, Qingqing Wang, Guohui Li, Dawei Li, Qufu Wei

**Affiliations:** 1 Key Laboratory of Eco-textiles, Jiangnan University, Wuxi 214122, Jiangsu, China; 6130703014@vip.jiangnan.edu.cn (P.L.); wqq888217@126.com (Q.W.); wykaojn@126.com (G.L.); ldw19900323@163.com (D.L.); 2 Key Laboratory of Textile Fabric, Anhui Polytechnic University, Wuhu 241000, Anhui, China; fengquan@ahpu.edu.cn

**Keywords:** bacterial cellulose, carbon nanotubes, laccase, glucose oxidase, enzyme biological fuel cell

## Abstract

Novel nanocomposites comprised of bacterial cellulose (BC) with carboxylic multi-walled carbon nanotubes (c-MWCNTs) incorporated into the BC matrix were prepared through a simple method of biosynthesis. The biocathode and bioanode for the enzyme biological fuel cell (EBFC) were prepared using BC/c-MWCNTs composite injected by laccase (Lac) and glucose oxidase (GOD) with the aid of glutaraldehyde (GA) crosslinking. Biosynthesis of BC/c-MWCNTs composite was characterized by digital photos, scanning electron microscope (SEM), and Fourier Transform Infrared (FTIR). The experimental results indicated the successful incorporation of c-MWCNTs into the BC. The electrochemical and biofuel performance were evaluated by cyclic voltammetry (CV) and linear sweep voltammetry (LSV). The power density and current density of EBFCs were recorded at 32.98 µW/cm^3^ and 0.29 mA/cm^3^, respectively. Additionally, the EBFCs also showed acceptable stability. Preliminary tests on double cells indicated that renewable BC have great potential in the application field of EBFCs.

## 1. Introduction

More attention has been increasingly paid to eco-friendly materials because of an increased awareness of sustainable development and environmental protection [1]. Nowadays, enzymatic biofuel cells (EBFCs) as the new green energy devices have drawn much attention because they are capable of harvesting electricity from renewable and abundantly available sources by using enzymes as the catalysts for oxidation of biofuels (most commonly, glucose) and reduction of oxidizers (most commonly, oxygen) [2,3]. They are renewable energy without any harmful intermediates and side products. Most EBFCs cathodes involve the four-electron reduction of O_2_ to water [4].

O_2_ + 4H^+^ + 4e^−^   →  2H_2_O (1)

Due to the fact that active centers of the enzymes are usually buried inside the protein matrix, electron transfer to the electrodes has been of crucial importance for EBFC performance [5]. Many types of carbon nanotubes (CNTs) have, therefore, been widely used as conductive nanowires in facilitating electron transfer from the catalytic centers of enzymes to the electrode surface because of their unique properties of being chemically inert with excellent conductivity, their electro-chemical stability, and molecular dimensions that enable intimate interaction with the enzymes [6,7,8]. These unique properties of CNTs make them extremely attractive for electrochemical applications, protein electrochemistry, electrochemical sensors, and especially for biosensors or biofuel [9,10]. In the bioelectronics field, CNTs have been used as supports for enzyme immobilization to enable direct electron transfer because of their large specific surface area and good conductivity [11].

Bacterial cellulose (BC), synthesized by Acetobacter xylinum, is a green, economical, and low-cost biopolymer [12]. As a general material, BC has distinctive properties including high ultrafine porosity, three-dimentional web-like structure, high crystallinity, water absorbance, mechanical properties, and biocompatibility, which makes it versatile in terms of application in many fields such as paper and paper-based products, audio components, tissue engineering, food, and electronic industries [13,14], as well as stretchable conductors [15], lithium ion battery anodes [16], conductive and fire-resistant aerogels [17]. Furthermore, the hydroxyl groups on its backbone can provide BC with a high hydrophilicity, which is crucial for the operation of polymer electrolyte membrane fuel cells [18]. However, these methods are not convenient to apply because they require additional time consuming, energy-intensive, or expensive steps, such as pyrolization at very high temperatures with the application of curing agents or chemical modification. Post-treatments have been carried out on cellulose to prepare a composite film of cellulose and reduced graphene for applications in supercapacitors [19], and flexible and conductive films [20].

**Figure 1:**
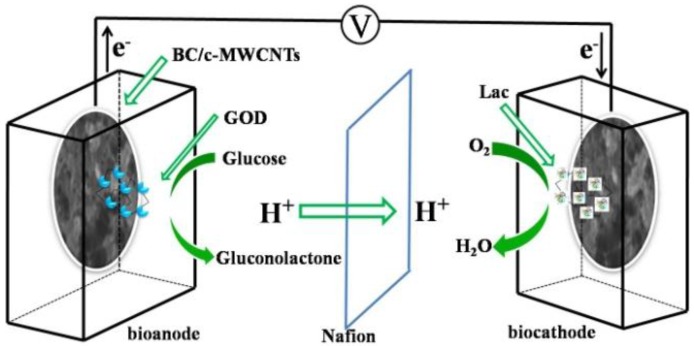
Illustration of the EBFC equipped with 3D BC/c-MWCNTs hybrid electrodes (not to scale).

In this work, biosynthesis of BC/carboxylic multi-walled carbon nanotubes (c-MWCNTs) in agitated culture were used as both bioanode and biocathode in EBFCs (Figure 1). The biocathode and bioanode were prepared and the BC/c-MWCNTs were injected with laccase (Lac) and glucose oxidase (GOD) by glutaraldehyde (GA) crosslinking, respectively. This new membrane electrode assemblies (MEAs) method offers theoretical and technological supports for exploiting high-efficient EBFCs.

## 2. Experimental Materials and Procedures

### 2.1. Chemicals

The industrial laccase powder (3 U/mg) from Trametes was purchased from Wuhan Nuohui Pharmaceutical And Chemical Co., Ltd. (Wuhan, Hubei, China); the industrial glucose oxidase (800 U/mg) was obtained Henan Huakang Chemical Co., Ltd. (Zhenzhou, Henan, China); the c-MWCNTs (OD, <8 nm; Length, 0.5–2 μm; Purity, >95%) was supplied by Nanjing XFNANO Materials Tech Co., Ltd. (Nanjing, Jiangsu, China), and glutaraldehyde was purchased from Henan Huakang Chemical Co., Ltd. (Zhenzhou, Henan, China).

### 2.2. Preparation of BC/c-MWCNTs Composite

First, c-MWCNTs were dispersed in culture media containing 2.5% (w/v) D-mannitol, 0.5% (w/v) yeast, 0.3% (w/v) bacto-peptone [21]. These culture media were sterilized at 120 °C in an autoclave for 2 h and poured into Erlenmeyer flasks. The bacterium was cultured on Hestrin and Schramm (HS) (5% (w/v) glucose, 1.6% (w/v) bacto-peptone, 0.2% (w/v) citric acid, 0.2% (w/v) disodium hydrogen phosphate, 0.3% (w/v) potassium dihydrogen phosphate, 0.03% (w/v) magnesium sulfate) medium [22] by static incubation. The pre-cultured cells in a test tube containing small cellulose particles on the surface of the medium were inoculated into a 100 mL Erlenmeyer flask containing 10 mL of the HS medium (in the presence of final 0.01 w/v% c-MWCNTs in culture media). The flasks were incubated on a rotary shaker operating at rotational speed of 100 rpm, for 6 days at 30 °C. The synthesized cellulose was separated from the medium by filtration and were dipped into 1% sodium hydroxide solution for 2 h at 80 °C in order to eliminate the cells and medium embedded in the cellulose material, then rinsed three times to pH 7 in deionized water [23].

### 2.3. Preparation of Enzyme Electrode

**Figure 2:**
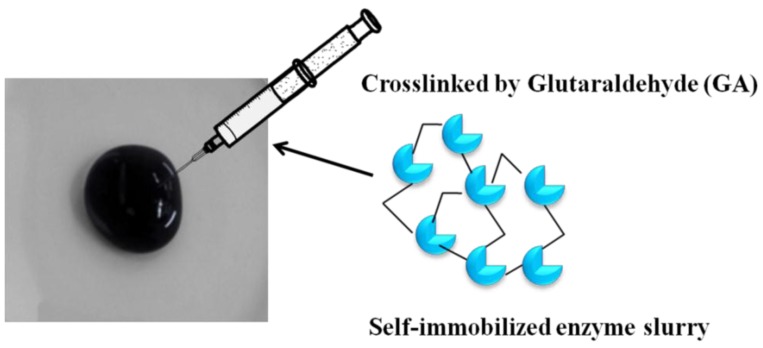
Illustration of the Lac and GOD immobilized on BC/c-MWCNTs by GA crosslinking.

The biocathode was prepared by the following method: BC/c-MWCNTs were injected by 40 mg mL^−1^ Lac with 1.5% (v/v) GA in 0.1 M acetic acid/sodium acetate buffer (pH = 5.5) solution for 1 h. The bioanode was prepared by similar method except that the 40 mg mL^−1^ Lac was replaced by 20 mg mL^−1^ GOD. Then, the electrode was dipped into 0.1 M acetic acid/sodium acetate buffer (pH = 5.5) solution containing 50 mM glucose, as shown in Figure 2.

### 2.4. Characterization and Electrochemical Measurements

The morphology of the BC and BC/c-MWCNTs composite membranes surfaces were characterized by field emission scanning electron microscope (FESEM, S-4800, Hitachi, Tokyo, Japan). The morphology of the BC was also characterized by high-resolution transmission electron microscope (TEM, JEOL/JEM-2100, Tokyo, Japan). Dried BC and BC/c-MWCNTs composite were placed over aluminum support and sputtered with gold. The samples were coated with a thin layer of Au nanoparticles to reduce the charging effects before FESEM observation.

The pure BC, c-MWCNTs, and BC/c-MWCNTs composite membranes were prepared in KBr pellet and scanned with Fourier transform infrared spectrophotometer (FTIR, Nicolet NEXUS, Hillsboro, OR, USA).

Electrochemical measurements were performed using a CHI 660D electrochemical workstation (CH Instruments, Inc., Austin, TX, USA). The electrochemical response was measured in a conventional three-electrode system using BC/c-MWCNTs/Lac and BC/c-MWCNTs/GOD as working electrode, a Pt wire auxiliary electrode and the Ag/AgCl as reference electrode. The electrocatalytic activity of bioanode material was tested toward oxygen reduction reaction in 0.1 M sodium acetate/acetic acid buffer solution (pH 5.5). The electrochemical measurements were carried out at around 25 °C.

## 3. Results and Discussion

### 3.1. Culture Process Characterization

**Figure 3:**
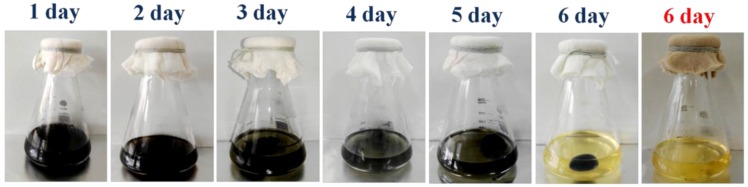
Growth of bacterial cellulose in the presence of c-MWCNTs (50 mg/100 mL) sheets: Photographic image of the six day track of bacterial cellulose growth in presence of c-MWCNTs under agitated conditions. The sixth day growth of bacterial cellulose in the absence of c-MWCNTs (red font).

Figure 3 illustrates a six-day track of bacterial cellulose growth in presence of c-MWCNTs under agitated culture. Firstly, *G. xylinus* was inoculated into the culture media contain c-MWCNTs. On the first day, culture solution fully presented black color as shown in Figure 3, but it could be seen that the color faded with the increasing number of days, revealing only a single black bacterial cellulose in the culture solution indicating the incorporation of c-MWCNTs into the bacterial cellulose. On the sixth day, the culture solution became clearer (similar to the original culture color), indicating the incorporation of remaining c-MWCNTs into BC pallet. The sixth day growth of bacterial cellulose in the absence of c-WMCNTs presented a white cryptomere structure.

**Figure 4:**
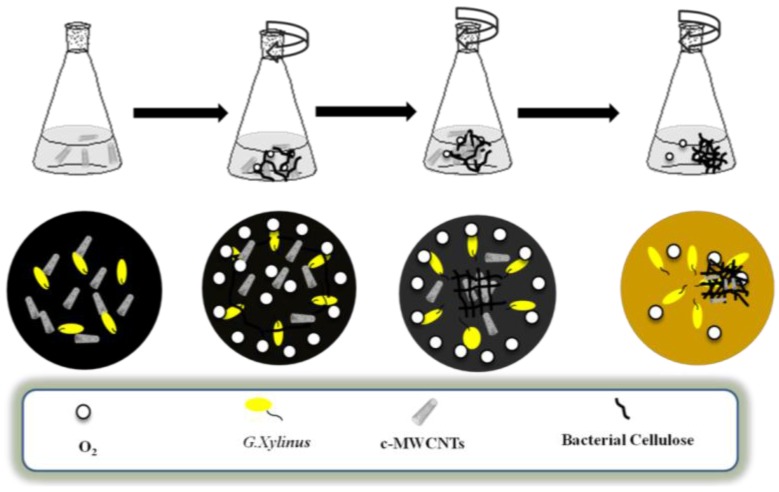
Schematic illustration of formation of bacterial cellulose and the integration of carbon into the 3D interconnected fibrous network of BC.

Figure 4 displays schematic illustration of the formation of bacterial cellulose and the integration of c-MWCNTs into the 3D interconnected fibrous network of BC. Firstly, c-MWCNTs were dispersed into culture solution and then the free bacteria was attached to the surface bubbles which apparently underwent reproduction to synthesize bacterial cellulose fibers [24]. As the days increased, the bacterial cellulose attached itself to the c-MWCNTs to form a more compact structure [25]. Due to the growth of the bacterial cellulose in the presence of c-MWCNTs under agitated conditions, the bacterial cellulose and c-MWCNTs entangled with each other and gradually gathered for the dispersion of c-MWCNTs in culture solution. It was again observed that due to the slow rate of BC-formation, c-MWCNTs was, therefore, absorbed on the BC whiles the bacteria gathered around the BC/c-MWCNTs composite and produced BC fibrils. The formation of new BC fibrils on existing composite was continuous until a more compact, irregularly shaped, and randomly overlapped composite was obtained.

### 3.2. Morphology Analysis

**Figure 5:**
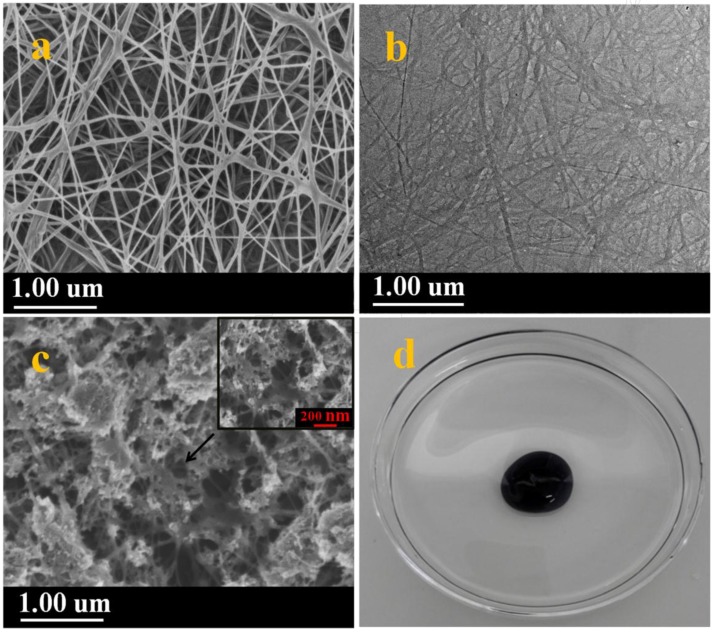
(**a**) FESEM images of pure BC synthesized in shaking culture system; (**b**) TEM image of BC; (**c**) FESEM images of BC/c-MWCNTs composite membranes; and (**d**) digital photos of c-MWCNTs-dispersed HS medium a shaking culture system at 30 °C for 6 days (100 rpm).

The microstructure of BC was characterized by scanning electron microscopy (SEM) and transmission electron microscopy (TEM). Figure 5a shows pure BC fibrils with ultrafine nanofiber structure, high ultrafine porosity 3D web-like structure. As shown in Figure 5a, BC revealed interconnecting pores which is in agreement with the report of refference [26]. The ultrafine network structure and larger specific surface area provided favourable channels for O_2_ transmission. The high-resolution TEM image further revealed that these nanofibers were mainly consisted of randomly orientated 3D web-like structure (Figure 5b) [27]. Figure 5c showed that c-MWCNTs was absorbed on 3D BC fibrils in which c-MWCNTs displayed the phenomenon of aggregation. As shown in Figure 5d, the morphology of BC/c-MWCNTs samples revealed that c-MWCNTs were tightly wrapped in BC pellicle resulting in an spherical shaped structure.

### 3.3. FTIR Analysis

**Figure 6:**
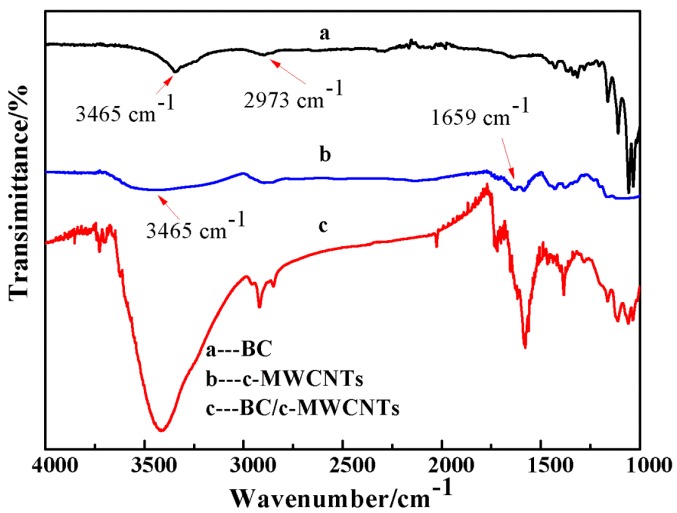
FTIR spectra of different composite. (**a**) BC; (**b**) c-MWCNTs; and (**c**) BC/c-MWCNTs composite.

Figure 6 shows the FT-IR spectra of the pure BC c-MWCNTs and BC/c-MWCNTs. The band at 3465 cm^−1^ for c-MWCNTs was attributed to the presence of hydroxyl groups(-OH) [28], as shown in Figure 6b. The absorption band at 1659 cm^−1^ for c-MWCNTs was assigned to the presence of carboxyl functional groups (C=O) which was in agreement with references [29,30]. The absorption band at 2973 cm^−1^ for BC（Figure 6a）was also attributed to the presence of C-H stretching vibrations which was in agreement with the characteristic bands of BC reported in the literature [31]. The main difference in the spectra of BC and BC/c-MWCNTs was found at the absorption peak of 3465 cm^−1^, as shown in Figure 6c. The peak at 3465 cm^−1^ for BC/c-MWCNTs composite membranes were enhanced, suggesting that only physical interaction occurred between BC and c-MWCNTs.

### 3.4. Effect of Different Conditions on Power Output of the EBFC

#### 3.4.1. The Influence of Different Glucose Concentrations

**Figure 7:**
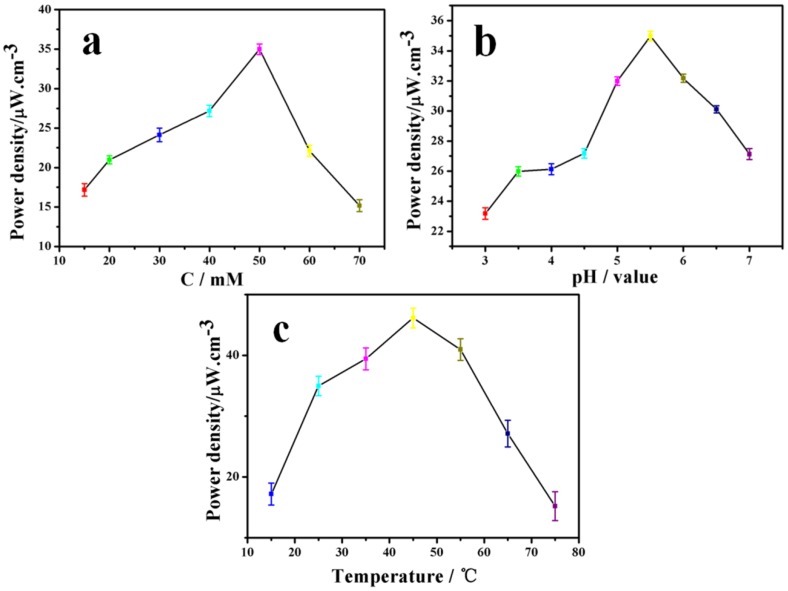
The maximum power output of the EBFC with different conditions: (**a**) glucose concentrations; (**b**) optimum pH; and (**c**) optimum temperature.

Figure 7a displays the maximum power output of the EBFC with different glucose concentration ranging from 15 mM to 70 mM. Figure 6a shows relatively higher power output in lower concentrations region (15–50 mM); however, when the glucose concentration further increased, the power output greatly decreased. In general, the power output showed a relatively higher enzyme activity among the range of 30–55 mM with an optimum concentration of about 50 mM.

#### 3.4.2. The Influence of Different pH

The biofuel cells were incubated in buffer solution with pH ranging from 3 to 7 at room temperature (Figure 7b). The immobilized laccase and glucose oxidase on BC/c-MWCNTs showed relatively higher pH stability. Additionally, the immobilized enzyme on BC/c-MWCNTs composite showed a relatively higher activity among the range of pH 4–6, and the optimum pH was around 5.5, as shown in Figure 7b.

#### 3.4.3. The Influence of Different Temperature

The effect of temperature on power density of immobilized enzyme on BC/c-MWCNTs is shown in Figure 7c. The immobilized enzyme was incubated in buffer solution (pH 5.5) for 5 min at different temperatures varying from 15 to 75 °C. Figure 7c shows relatively higher activity retention in lower temperature region (30–50 °C), however when the temperature further increased, power output greatly decreased indicating the decrease of enzyme activity. The immobilized enzymes showed a higher enzyme activity among 45–60 °C with an optimum temperature at 55 °C. In general, the immobilized enzyme possessed high activity in a broader temperature range, enabling it a desirable material for enzymatic biofuel cells application and many other fields.

### 3.5. Electrochemical Behavior of the Lac/BC/c-MWCNTs Electrode

**Figure 8:**
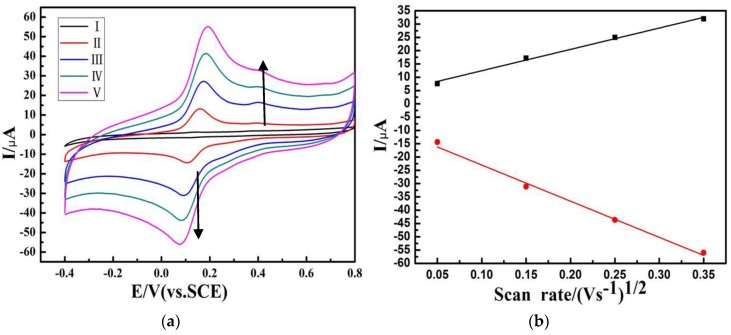
(**a**) Cyclic voltammograms of (I) the bare 3D BC/c-MWCNTs electrode and BC/c-MWCNTs/Lac electrode in a 0.1 M acetic acid/sodium acetate buffer solution (pH 5.5) at scan rates (mV s^−1^): (II) 50, (III) 150, (IV) 250, (V) 350; and (**b**) The inset is a plot of the oxidation and reduction peak currents *vs.* the scan rates.

As shown in Figure 8a, the CVs of the BC/c-MWCNTs/Lac electrode were absent, whereas a pair of prominent redox peaks (at 0.74 V and 0.11 V, respectively) can be observed [32], which was attributed to the redox reaction of the Lac immobilized on the c-MWCNTs. This showed the good coupling between the enzymes and 3D BC/c-MWCNTs substrate. The redox of Lac on the electrode was a reversible and surface-confined process, which could be demonstrated by the linear relationship between redox currents and scan rate, as shown in Figure 8b. The bare 3D BC/c-MWCNTs electrode showed no catalytic action to O_2_ in comparison with that of BC/c-MWCNTs/Lac electrode.

### 3.6. Performance of the Biofuel Cell

**Figure 9:**
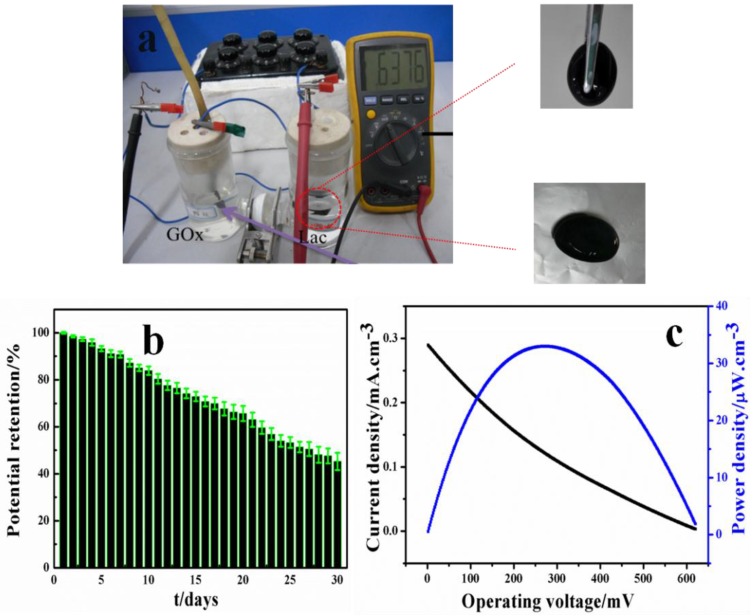
(**a**) The maximum open circuit voltage of the EBFCs; (**b**) The open circuit voltage from the EBFCs over 30 days; and (**c**) The power density curve of glucose/O_2_ biofuel cell obtained by LSV in 0.1 m, pH 5.5, HAc/NaAc buffer containing 50 mM glucose, data were collected at 10 mV s^−1^.

The EBFCs were fabricated with a 3D BC/c-MWCNTs/Lac cathode and 3D BC/c-MWCNTs/GOD anode by GA crosslinking. As demonstrated in Figure 9a, the $E_{\mathrm{cell}}^{\mathrm{ocv}}$ of the EBFC was approximately 0.64 V. Through 30 days of open circuit voltage collected, a 51% decrease of open circuit voltage was observed indicating relatively satisfactory stability, as shown in Figure 9b. Linear sweep voltammetry (LSV) was used to evaluate the electrochemical performance of enzymatic biofuel cell operating with glucose [33]. As shown in Figure 9c, the change of power density showed first increase and then a decrease with the decline of open circuit voltage whiles the open-circuit voltage was about 0.62 V which was relatively consistent with the biggest open circuit voltage in Figure 9a. The maximum current density was 0.29 mA/cm^3^ and the maximum power density was 32.98 µW/cm^3^ implying acceptable electric properties.

## 4. Conclusions

Lac and GOD were respectively immobilized on the BC/c-MWCNTs composite to prepare the cathode and anode of EBFCs. The nanotopographic surface of c-MWCNTs networks ensured snug anchoring of enzyme molecules by GA crosslinging. The influence of different concentrations, pH and temperature on the performance of EBFCs was investigated, and the optimum concentration, pH and temperature were 50 mM, 5.5, and 55 °C, respectively. The as-prepared EBFCs showed satisfactory electric properties. Our study demonstrated that the three-dimensional structure, controllable porosity as well as designable shape of BC could provide the application possibility in biofuel cell fields.
